# Estimation of carbon stocks in wood products for private building companies

**DOI:** 10.1038/s41598-022-23112-0

**Published:** 2022-10-27

**Authors:** Ryoto Matsumoto, Chihiro Kayo, Satoshi Kita, Kentaro Nakamura, Christian Lauk, Ryo Funada

**Affiliations:** 1 Graduate School of Agriculture, Tokyo University of Agriculture and Technology, 3-5-8 Saiwai-cho, Fuchu, Tokyo 183-8509 Japan; 2 Forest and Landscape Research Center, Sumitomo Forestry Co., Ltd., Keidanren Kaikan, 1-3-2 Otemachi, Chiyoda-ku, Tokyo 100-8270 Japan; 3 Institute of Social Ecology, Department of Economics and Social Sciences, University of Natural Resources and Life Sciences Vienna, Schottenfeldgasse 29, 1070 Vienna, Austria

**Keywords:** Climate-change mitigation, Environmental economics, Climate-change mitigation, Sustainability

## Abstract

Wood products function as carbon storage even after being harvested from forests. This has garnered attention in relevance to climate change countermeasures. In the progress of efforts toward climate change mitigation by private companies, the effective use of wood products has been an important measure. However, the methodology for accounting carbon stocks in wood products for private companies has not been established. Therefore, this study investigated methods for estimating carbon stocks in wood products used in wooden houses built by private enterprises, targeting a major company in the Japanese building industry. The results indicated that both the direct inventory method and flux data method (FDM) were applicable for estimating the carbon stocks. These two methods use data that can be obtained from many other building companies, thus, indicating high versatility. The log-normal, Weibull, normal, and logistic distributions, in descending order, proved to be suitable lifetime functions of wooden houses under the FDM, with a half-life of 66–101 years. It is important to continuously acquire time-series data on the floor areas of both newly built and existing houses and the amount of wood products used to improve the accuracy of estimates and explore future predictions.

## Introduction

Combating climate change is one of humanity’s biggest challenges, and it is crucial to make rapid progress to reduce the quantity of greenhouse gases (GHGs) and increase their removal globally^1^. Wood can store carbon even after being harvested from forests, thereby impacting the global carbon cycle. This function of wood has been identified as a climate change countermeasure^2–7^.

Countries are required to account and report the annual change (i.e., emission and removal) in carbon stocks in harvested wood products in their national GHG inventory to the United Nations Framework Convention on Climate Change^8^. Harvested wood products are also used for nationally determined contributions under the Paris Agreement^9^. Accordingly, carbon stocks in wood products have been estimated mainly at the national scale^10–16^. As for Japan, these estimates have been conducted at the national or prefectural scales^17–21^.

At the same time, the commitments of private companies to climate change mitigation, including Science Based Targets^22^, Renewable Energy 100^23^, and Task Force on Climate-related Financial Disclosures^24^, have been substantially important. In this context, the GHG Protocol has provided global standards and guidance to estimate and report GHG emissions for private companies^25^.

While the efforts of private corporates have hitherto mainly focused on GHG emission reductions, carbon removal and storage in forests and wood products are drawing more attention recently^26^. Accordingly, the GHG Protocol has begun to develop the standard and guidance on carbon accounting for land use, land use change, and biogenic products, but these standards are still under development^26^. In Japan, Forestry Agency has published guidelines for private companies to indicate the amount of carbon storage in wood used in buildings^27^. However, the guidelines treat wood products used in a newly constructed building as carbon stocks, which are just carbon inflows to existing carbon stock pools, not carbon stocks as such. Consequently, the methodology and framework for accounting carbon stocks in wood products for private companies have not been established.

Therefore, focusing on the building industry, which has the largest carbon stocks among all wood-related industries^5,6,28,29^, the objective of this study was to investigate methods for estimating carbon stocks in wood products used in wooden houses built by a major building enterprise in Japan and then estimate the carbon stocks. Furthermore, we discussed the data and methods requirements to improve the accuracy of the estimates.

## Results

We found that carbon stocks in wood products used in wooden houses built by the target company, Sumitomo Forestry Co., Ltd., could be estimated using the direct inventory method (DIM) and flux data method (FDM)^30^. While the DIM directly determined the amount of carbon stock by using the number of existing wooden houses, the FDM indirectly estimated the amount of the carbon stock by using the number of wooden houses annually built and a lifetime function, whose parameter is the half-life of wooden houses (see Methods section).

Carbon stocks in wood products used in existing wooden houses were estimated to be approximately 1.96 million t-C at the start of fiscal year (FY) 2021 under the DIM, which are equivalent to 53% of those (approximately 3.68 million t-C) in forests (approximately 48,000 ha) owned by the target company^31^. The results indicate that wooden houses function as marked carbon storage.

**Figure 1:**
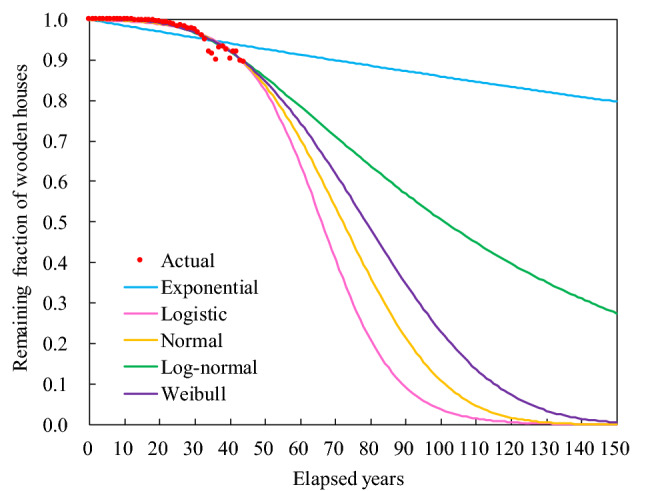
Estimated lifetime functions of wooden houses. *Note*: “Actual” indicates the actual remaining fraction calculated based on the number of existing and built wooden houses (see the “Flux data method” section). “Exponential,” “Logistic,” “Normal,” “Log-normal,” and “Weibull” represent the estimated remaining fraction based on parameters that minimize the residual sum of squares (RSS) between the actual values and the estimated values for each lifetime function.

Figure 1 shows the estimated lifetime functions based on parameters that minimized the residual sum of squares (RSS) between the actual values of the remaining fraction of wooden houses and their estimated values under the FDM. In addition, Table 1 lists their parameters and RSS. For exponential distribution (first order decay, FOD), the minimal RSS was estimated to be 0.02, representing the largest in all lifetime functions, and the estimated half-life value was 459 years, which was abnormally long. The other four lifetime functions had the least RSS of 0.004–0.006 and half-life values ranging from 66 to 101 years. Log-normal and Weibull distributions particularly provided the minimal RSS, which can be considered as suitable lifetime functions for wooden houses.

**Table 1:** Parameters of lifetime functions of wooden houses.

	Exponential	Logistic	Normal	Log-normal	Weibull
Half-life (years)	459	66	72	101	79
Parameter *α*	–	0.09555	22.60593	0.66116	3.14451
Parameter *β*	–	–	–	–	88.35761
RSS	0.02091	0.00644	0.00565	0.00449	0.00498

The half-life values and the other parameters correspond to the estimations shown in Fig. 1. “RSS” denotes the least residual sum of squares between the actual and estimated values for each lifetime function.

**Figure 2:**
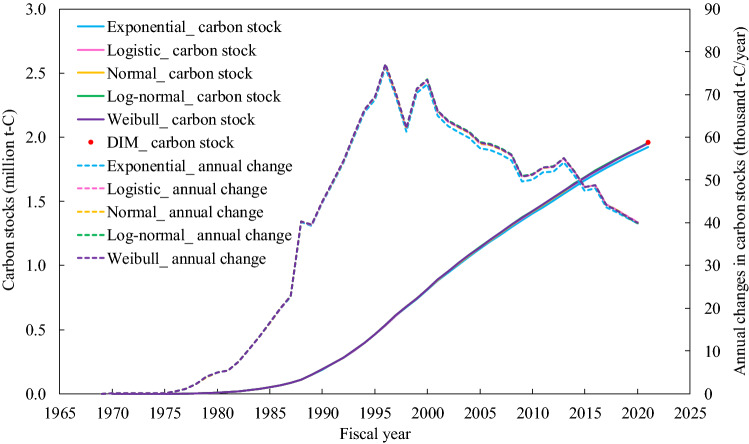
Estimated carbon stocks in wood products used in existing wooden houses. *Note*: “DIM” indicates the estimated results using the direct inventory method (DIM). Each lifetime function denotes the estimated results based on the parameters in Fig. 1 and Table 1 using the flux data method (FDM).

Figure 2 shows the estimated carbon stocks and their annual changes in wood products used in existing wooden houses by adopting each lifetime function with parameters that minimized the RSS. Differences from the estimated carbon stocks (1.96 million t-C) at the start of FY2021 for the DIM were 1.52%, 0.27%, 0.17%, 0.01%, and 0.08% in exponential, logistic, normal, log-normal, and Weibull distributions, respectively, for the FDM. The carbon stocks continued to increase since FY1969 under all lifetime functions. The annual changes in carbon stocks increased until 1996, but the annual increase showed a decreasing trend thereafter.

**Figure 3:**
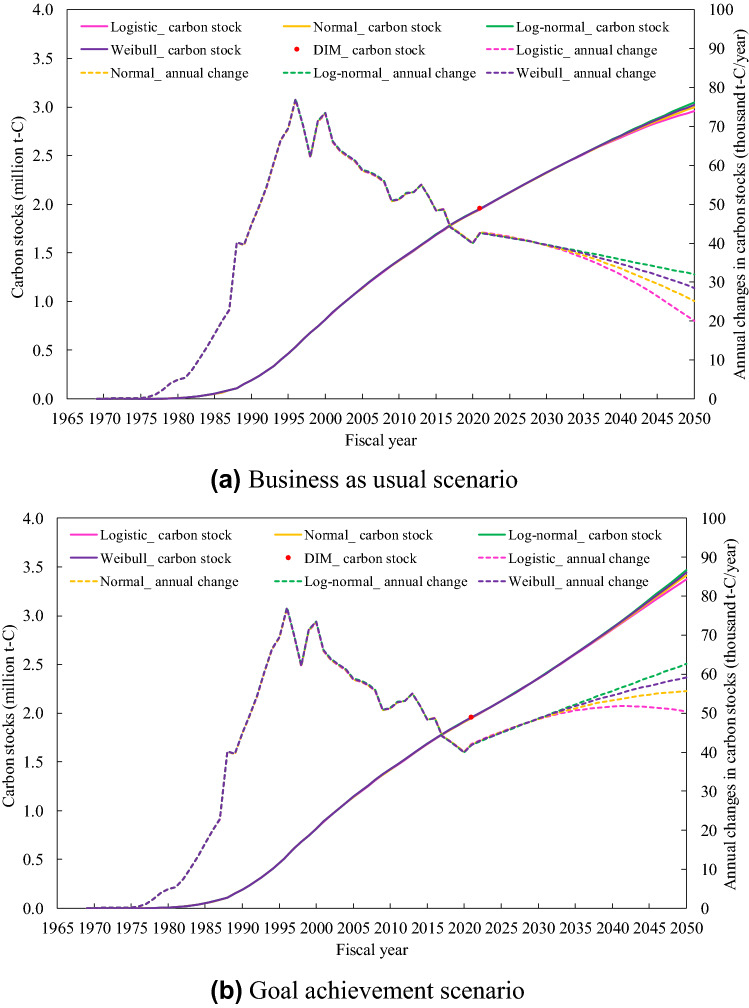
Future prediction of carbon stocks in wood products used in existing wooden houses until FY2050. (**a**) Business as usual scenario: The yearly average number of wooden houses built for the past five years during FY2016–FY2020 (8348 houses/yr) was assumed to continue to FY2050. (**b**) Goal achievement scenario: The target company’s goal of the number of domestic wooden houses built in FY2030 (10,000 houses/yr) was assumed to be attained and it continued with the same increasing trend till FY2050.

The FDM enabled future predictions of carbon stocks in wood products based on future scenarios related to the demand for new wooden housing and changes in house lifetime, which contribute to the investigation of future goals and measures. Figure 3 shows future predictions of the carbon stocks until FY2050 using the four lifetime distributions (except for the exponential), which showed suitable lifetime functions. Under the business as usual scenario, which maintains the yearly average number of wooden houses built for the past five years during FY2016–FY2020 to FY2050, the carbon stocks in FY 2050 were estimated to be approximately 2.96–3.05 million t-C (Fig. 3a). Furthermore, in the goal achievement scenario, in which the goal of the number of wooden houses to be built in FY2030 (10000 houses/yr) will be achieved by the target company, the estimated carbon stocks in FY2030 and FY2050 will be approximately 2.36 million t-C and 3.37–3.47 million t-C, respectively (Fig. 3b). For both scenarios, there were considerable differences in annual changes in carbon stocks among the four lifetime functions. The maximum difference in the annual changes among them in FY2050 was 38% between the logistic and log-normal distributions for the business as usual scenario (Fig. 3a) and 19% for the goal achievement scenario (Fig. 3b).

## Discussion

At the start of FY2021, carbon stocks in wood products used in existing wooden houses built by the target company (see Fig. 2) were equivalent to more than half of the carbon stocks in forests owned by the company, based on the DIM, thus, revealing that wooden houses store a notable amount of carbon.

For the FDM, the exponential distribution (FOD), as suggested in the Intergovernmental Panel on Climate Change (IPCC) guidelines^32,33^ had an inordinately long half-life and large RSS (see Table 1), which cannot be an appropriate lifetime function for Japanese wooden houses. Meanwhile, among the other lifetime functions, the log-normal and Weibull distributions showed the least RSS (see Table 1) and were inferred to be suitable lifetime functions. The exponential distribution^3,28,32,33^ and the normal distribution^34–37^ are globally well-known functions. Conversely, previous studies from Japan focusing on houses in major cities^38,39^ and wooden buildings at the national level^40^ have favored the log-normal and Weibull distributions and reported that the log-normal distribution was particularly suitable for wooden houses. This corresponded with our results. However, because there is little difference in the RSS between the logistic/normal distributions and the log-normal distribution (see Table 1), both these functions can be considered as appropriate lifetime functions depending on future transitions of the remaining fraction of wooden houses (see Fig. 1).

The yearly half-life values of wooden housing in major cities in Japan were reportedly in the range of around 30–50 years between the 1980s and the early 2000s^38,39,41,42^. The half-life of wooden buildings throughout Japan was reported to be 63 years between 1997 and 2020^40^. Conversely, in this study, the half-life of wooden housing in the target company was found to be 101, 79, 72, and 66 years (see Table 1) for the log-normal, Weibull, normal, and logistic distributions, respectively, which was considerably longer than the values reported in previous studies^38–42^. Internationally, studies have reported the half-life values of buildings and housing to be 71 years in Austria^43^, 80 years in the United Kingdom^3,37^, 61–100 years in the United States^3,44,45^, 125 years in Norway^34^, and 65–150 years in Germany^3,36^, indicating that wooden house lifetimes by the target company had similar half-life values to those in the United States and European countries.

**Figure 4:**
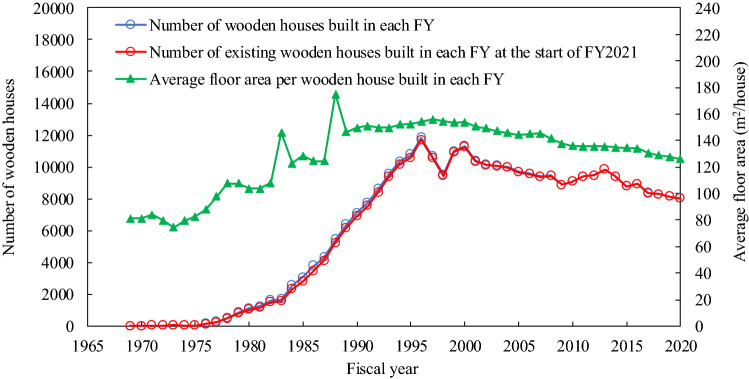
Number of wooden houses and average floor area per house built by the target company.

By inspecting the carbon stocks in wood products used in wooden houses at the start of FY2021 (see Fig. 2), we found that the estimated values in the lifetime functions other than the exponential function were almost consistent with the actual values (the difference was less than 0.3%). Specifically, the log-normal and Weibull distributions had the least difference of less than 0.1% between their estimated and actual values, suggesting that the estimates had the highest accuracy. While the annual changes in the carbon stocks continued to be positive until 1996 (annual increases), the annual increases had a decreasing trend after 1997. Japanese society witnessed steady economic growth and an associated increase in housing demand between the 1960s and the early 1990s (Fig. 4), which led to increases in annual changes in the carbon stocks. However, the economic conditions have worsened since the 1990s^46^. Moreover, 1997 saw an increase in the consumption tax^47^, and this resulted in a further recession hindering the construction of new houses and reducing the annual increases in the carbon stocks.

While the estimated values of carbon stocks under all of the four lifetime functions were in good agreement with the actual value in FY2021 (Fig. 2), the longer the period of future predictions was assumed, the larger the differences in annual changes in carbon stocks among the four functions occurred (Fig. 3). This indicates that accurate estimation of carbon stocks and their annual changes requires the determination of the appropriate lifetime function.

We revealed that the carbon stocks in existing wooden houses at the start of FY2021 can be estimated using the DIM based on data owned by the target building company. Obtaining data on the number of existing wooden houses in a year enabled this estimate. However, there is a possibility that the standards and guidelines for private companies, such as the GHG Protocol, require measurements and reports of not only the amount of carbon stock but also their annual changes in accordance with the IPCC guidelines^32,33^. In addition, it is important to understand the historical transitions of carbon stocks. Therefore, surveys on the numbers of existing wooden housing should be conducted every year, instead of only individual years.

In contrast, the carbon stocks in wooden houses and their annual changes for the past half-century can be estimated using the FDM. This became possible due to the acquisition of data on the number of wooden houses built every FY. Simultaneously, this method needs the use of a lifetime function which requires the identification of a suitable function type and parameters including half-life. Since we successfully determined the remaining fraction of wooden houses for years elapsed after construction by obtaining data on the number of existing wooden housing built in each FY at the start of FY2021, this enabled the estimation of parameters of the lifetime function. Thus, to improve the accuracy of the carbon stock estimates under the FDM, it is important to arrive at the number of existing wooden houses as with the DIM.

While the DIM is considered to provide a highly accurate estimation of carbon stocks because it directly uses the number of existing wooden houses which indicates wooden houses stock, acquisition of time-series data on the existing houses is not easy even for a building company. Meanwhile, the FDM is expected to be relatively readily available since it uses the number of newly built wooden houses which shows annual wooden houses inflow and is relatively easily obtained, whereas it requires an assumption of lifetime function types and half-life values. Considering that the FDM can estimate both historical transitions and future predictions of the carbon stocks by using the number of newly built houses which can be obtained more easily than the number of existing houses required in the DIM, the FDM is more advantageous than the DIM. Furthermore, for the FDM, the suitable lifetime function can be determined by using the methodology proposed in this study.

Both DIM and FDM used data on the average floor area per wooden house to convert the number of wooden houses to their floor areas. It is preferable for the estimates which reflect the realities to use floor area for each wooden house rather than the average floor area because the floor area varies depending on the house.

Since the data that we could obtain on the amount of wood products used in yearly built wooden houses were only for two years (FY2018 and FY2019), we applied the average amount of wood products used per unit floor area of the two years to the whole target period. However, the amount of wood products used in wooden houses is expected to differ according to the year, and thus, a yearly survey on the amount of wood products is desirable.

Converting the amount of wood products to their carbon amount requires the density and carbon content of wood, but it is not easy to obtain the density values for each tree species. Air dry density (air dry mass over air dry volume) and basic density (oven dry mass over green volume) have been often published for many tree species^21,32,48,49^. On the contrary, literature on the density values, which represent oven dry mass over air dry volume and are essential to determine carbon stocks in wood products, is largely lacking^32,33^. Further, there is no published database for Japanese tree species. Therefore, it is important to establish a database with density values with oven dry mass over air dry volume.

The limitation of this study is that for the determination of an appropriate lifetime function, we did not consider the possibility of changing lifetime function types and half-lives during the target period between FY1969 and FY2020. Although we assumed that the lifetime function type and half-life of wooden houses have not changed over the last 50 years, the previous study^40^ reported that the half-life of wooden buildings throughout Japan has been prolonged over time. Obtaining data on house stock at multiple time points is necessary to consider this for future research. Moreover, this study targeted wooden houses, but the suitable lifetime function type and half-life values could differ when other building types such as non-wooden offices, stores, and factories are targeted. This is also a limitation of this study.

## Conclusions

This study examined methods for estimating carbon stocks in wood products used in existing wooden houses built by private building companies and estimated these values focusing on wooden houses built by Sumitomo Forestry Co., Ltd. Moreover, we discussed the data and methods required for more accurate estimates.

Applicability of the DIM and FDM, which were used in the IPCC guidelines and Japanese National Inventory Report (NIR), was examined. Consequently, both methods were found applicable using data on the number of wooden houses built in each FY, the number of existing wooden houses built in each FY at the start of FY2021, the average floor area per house built in each FY, and the amount of wood products for each tree species used in wooden houses. Accordingly, we estimated the amount of carbon stock in a single year using the DIM and that in each year for the past half-century using the FDM. Above mentioned data are expected to be available with or are possible to be collected by most building companies, and thus, the methods proposed in this study are applicable to many other companies, thereby suggesting their high versatility.

Carbon stocks in wood products used in existing wooden houses at the start of FY2021 were estimated to be approximately 1.96 million t-C under the DIM, equal to more than half of carbon stocks in domestic forests owned by the target company, which suggests that wooden houses play a significant role in carbon storage.

Further, we determined the lifetime function with parameters that minimized the RSS between the estimated values and the actual values of the remaining fraction of wooden houses. The log-normal, Weibull, normal, and logistic distributions, in descending order, were suitable lifetime functions of wooden houses while the exponential distribution was not suitable under the FDM. In addition, the estimated half-life values for these lifetime functions were between 66 and 101 years, suggesting that wooden houses in the target company have longer lifetimes than those reported in previous studies from some major Japanese cities.

It is important for private building companies to continuously acquire time-series data on the floor areas of both newly constructed and existing houses and wood products used in them to improve the accuracy of estimates in the future.

Currently, frameworks for accounting of carbon stocks in wood products for private companies have not been established, whereas there are previous studies on carbon stocks in wood products at the national and regional scales. Although this study targets one building company, our proposed methods use widely obtainable data for many other companies. Therefore, the findings obtained in this study can contribute to the understanding of the status of carbon stocks in wood products and examine goals and measures based on their future predictions for other private companies. The methods and models proposed in this study are useful for scientifically quantifying carbon stocks in actual business activities by companies in the context of the GHG Protocol and enable the dissemination of contribution to climate change measures to society widely in their corporate social responsibility.

## Methods

### Target company, buildings, and wood products

We targeted wood products used in wooden houses built by Sumitomo Forestry Co., Ltd., a major building company in Japan. A wooden house is a structure and application with the largest share of constructed floor area^50^ and wood use amount per unit floor area^51^ in Japanese buildings. Sumitomo Forestry is one of the largest house seller companies in Japan^52^ and has a long history of over 50 years in the wooden house business^53^. As they were expected to hold substantial data on wood products used in wooden houses, we judged the company as an appropriate case study.

### Data

Sumitomo Forestry Co., Ltd. owns four data types on wood products used in wooden houses: (1) number of wooden houses built in each FY between FY1969 and FY2020 (e.g., April 1, 1969 to March 31, 1970) (Fig. 4, unit: house/yr), (2) number of existing wooden houses built in each FY between FY1969 and FY2020 at the start of FY2021 (Fig. 4, unit: house), (3) average floor area per house built in each FY between FY1969 and FY2020 (Fig. 4, unit: m^2^/house), and (4) amount of wood products for each tree species used in wooden houses built in FY2018 and FY2019 (unit: m^3^/yr). The wooden houses were all detached residences built with a wooden framework method. Supplementary Tables S1 and S2 show the numerical data (1)−(3) and (4), respectively.

### Estimation methods

Carbon stocks in wood products can be estimated using the FDM and DIM^30^. The FDM indirectly estimates the volume of existing wood products (m^3^) by multiplying annual wood product inflow (m^3^/yr) by a lifetime function, whose parameter is the half-life of wood products, and then converts the volume to the carbon amount (t-C). This method was used in Tiers 1 and 2 and a part of Tier 3 in the 2006 and 2019 guidelines provided by the IPCC^32,33^. It is also used in the Japanese NIR^21^ to estimate the annual changes in carbon stocks of wood products that are not used in building construction but in applications, such as furniture and paper/paperboard. In contrast to FDM, the DIM directly determines the volume of existing wood products (m^3^) and converts it into the carbon amount (t-C). The method is used in another part of Tier 3 in the IPCC guidelines^32,33^ and the Japanese NIR^21^ for wood products in buildings. While the FDM is commonly used in various countries and wood applications worldwide because it uses annual wood product inflow data that are more readily available, it requires an assumption of lifetime function types and half-life values. Contrastingly, the DIM is expected to be highly accurate since it directly uses the existing volume of wood products without the need for assumptions of lifetime functions. However, directly obtaining the existing volume is difficult, and thus, the method is only applicable in limited wood applications, such as buildings^32,33^. This study investigated the feasibility of the two methods in estimating carbon stocks in wood products used in wooden houses built by the target company.

#### Direct inventory method

We found that the DIM can be used to estimate carbon stocks in wood products used in existing wooden houses at the start of only FY2021 using the above data and determined them using Eq. (1).

1 $$ CSD_{i,j} \left( {t_{2021} } \right) = S_{i} \left( {t_{2021} } \right) \cdot A_{i} \cdot W_{j} \cdot D_{j} \cdot C $$

where $${ }CSD_{i,j} \left( {t_{2021} } \right)$$ (t-C) indicates the amount of carbon stock in wood products for tree species $$j$$ used in existing wooden houses built in FY*i* at the start of FY2021 under the DIM. $$S_{i} \left( {t_{2021} } \right)$$ (houses) represents the number of existing wooden houses built in FY*i* at the start of FY2021. $$A_{i}$$ (m^2^/house) indicates the average floor area per wooden house built in FY*i*.$$ W_{j}$$ (m^3^/m^2^) indicates the amount of wood products for tree species $$j$$ used per unit floor area. $$D_{j}$$ (t/m^3^) represents the density of wood (oven dry mass over air dry volume) for tree species $$j$$. $$C$$ (t-C/t) indicates the carbon content of oven-dried wood. Furthermore, $$i$$ is each FY between FY1969 and FY2020.

The number of existing wooden houses $\left( {S_{i} \left( {t_{2021} } \right)} \right)$ and the average floor area per wooden house $\left( {A_{i} } \right)$ were obtained from data (2) and (3) (Fig. 4 and Supplementary Table S1), respectively, in the Data section.

The amount of wood products used per unit floor area $\left( {W_{j} } \right)$ was calculated using the above data (1), (3), and (4) (Supplementary Tables S1 and S2). The floor area of wooden houses built in FY2018 and FY2019 (m^2^/yr), respectively, was determined by multiplying the number of wooden houses built in FY2018 and FY2019 (data (1) in the Data section) by the average floor area per wooden house in the same FYs $\left( {A_{i} } \right)$ (data (3) in the Data section). Following this, the amount of wood products for tree species *j* used per unit floor area in FY2018 and FY2019 (m^3^/m^2^), respectively, was obtained by dividing the amount of wood products for each tree species used in wooden houses built in the same FYs in the above data (4) by the above floor area of wooden houses built in the same FYs. The average of the amount of wood products used per unit floor area in FY2018 and that in FY2019 (0.218 m^3^/m^2^) was used for the whole target period during FY1969–FY2020. For more accurate estimation, using the amount of wood products for each FY is preferable. However, we used the above average amount value since the target company owned it only for the two FYs.

The density of wood (oven dry mass over air dry volume) for each tree species $\left( {D_{j} } \right)$ was determined based on the literature^27,48^. Air dry density (i.e., air dry mass over air dry volume) values, which include approximately 15% moisture content, have been provided in the literature. Therefore, the density in oven dry mass over air dry volume was obtained by multiplying the above air dry density in air dry mass over air dry volume^27,48^ by 100/115; the wood density is shown in Table 2. The carbon content of oven-dried wood ($C$) was assumed to be 0.5 for all tree species.

**Table 2:** Wood density (oven dry mass over air dry volume).

Tree species/wood product category	Density (t/m^3^)
Japanese cedar	0.331
Hinoki cypress	0.383
Japanese red pine	0.452
Japanese larch	0.435
Sakhalin fir	0.348
Yezo spruce	0.374
Douglas fir	0.479
Western hemlock	0.400
Norway spruce	0.374
Sequoia sempervirens	0.400
Mixture of Norway spruce and Sequoia sempervirens	0.387
Mixture of Douglas fir and Dahurian larch	0.457
Mixture of lodgepole pine, white spruce, and noble fir	0.395
Plywood, wood board, laminated veneer lumber	0.398

#### Flux data method

We found that the FDM is also applicable in estimating carbon stocks in wood products used in existing wooden houses and determined them using Eqs. (2) and (3). Additionally, the annual change in the carbon stocks was determined by Eq. (4).

2 $$ CSF_{i,j} \left( t \right) = \mathop \sum \limits_{{i = t_{1969} }}^{t - 1} \left[ {IF_{i,j} \cdot \left( {1 - L\left( {t - 1 - i} \right)} \right)} \right] $$

3 $$ IF_{i,j} = B_{i} \cdot A_{i} \cdot W_{j} \cdot D_{j} \cdot C $$

4 $$ ACF_{i,j} \left( {t - 1} \right) = CSF_{i,j} \left( t \right) - CSF_{i,j} \left( {t - 1} \right) $$

where $CSF_{i,j} \left( t \right)$ (t-C) indicates the amount of carbon stock in wood products for tree species $j$ used in existing wooden houses built in FY*i* at the start of FY*t* under the FDM. $IF_{i,j}$ (t-C/year) represents the amount of carbon inflow to the carbon stock pool in wood products for tree species $j$ used in wooden houses built in FY*i*. $L\left( {t - 1 - i} \right)$ represents the wooden house lifetime function (cumulative distribution function of wooden house lifetime distribution) for the years elapsed from FY*i* to FY*t* − 1^40^. Therefore, $1 - L\left( {t - 1 - i} \right)$ shows the remaining fraction of wooden houses for the years elapsed ($t - 1 - i$), and half-life is defined as the number of years passed in which the remaining fraction attains 0.5. $B_{i}$ (house/year) indicates the number of wooden houses built in FY*i*. $ACF_{i,j} \left( {t - 1} \right)$ (t-C/year) represents the amount of annual change in carbon stocks in wood products in wooden houses during FY*t* − 1. Furthermore, $t_{1969}$ is FY1969 and *t* is each FY until FY2021.

The number of wooden houses built ($$B_{i}$$) was obtained from data (1) (Fig. 4 and Supplementary Table S1) in the Data section. The average floor area per wooden house ($$A_{i}$$), amount of wood products used per unit floor area ($$W_{j}$$), density of wood ($$D_{j} )$$, and carbon content ($$C$$) were the same values as those used in the abovementioned DIM estimation (see Eq. (1)).

Regarding the lifetime function ($L\left( {t - 1 - i} \right)$), in addition to exponential distribution, which represents FOD suggested in the IPCC guidelines^32,33^, Japanese NIR^21^, and previous research^3,28^, we examined normal^34–36,38,40,54^, log-normal^37–42,44^, logistic^17,18,40^, and Weibull^34,37,40,41^ distributions.

We determined the parameters including a half-life that minimize the RSS between the actual and estimated values of the remaining fraction of wooden houses ($1 - L\left( {t - 1 - i} \right)$) for each lifetime function. The actual value of the remaining fraction of wooden houses for the elapsed years (“Actual” in Fig. 1) was determined by dividing the number of existing wooden houses built in FY*i* at the start of FY2021 ($S_{i} \left( {t_{2021} } \right)$) by the number of wooden houses built in FY*i* ($B_{i}$). The elapsed years indicate $t_{2021} - 1 - i$. $t_{2021}$ is FY2021 and $i$ is each FY between FY1976 and FY2020. According to an interview with the target company, the data on existing and built wooden houses between FY1969 and FY1975 in the early period of building business did not ensure their reliability. Moreover, the number of wooden houses built in the initial seven years was only 0.07% of the total number of wooden houses of the entire target period. Therefore, we judged that the data for the initial seven years were not suitable for estimating the remaining fraction and excluded them from the estimates.

### Future prediction

As mentioned above, since the FDM estimates the amount of carbon stocks using the wood products inflow ($IF_{i,j}$ in Eq. (2)) and the lifetime function ($L\left( {t - 1 - i} \right)$ in Eq. (2)), future predictions of the amount of carbon stock can be conducted by setting future scenarios and goals on the number of wooden houses built and their lifetime. As an example, future predictions of the carbon stocks until FY2050 were conducted using the number of wooden houses built and the lifetime functions. We set two future scenarios: “business as usual scenario” and “goal achievement scenario.” The business as usual scenario was assumed to maintain the yearly average number of wooden houses built during the period of FY2016–FY2020 ($B_{i}$ in Eq. (3), 8348 houses/yr) to FY2050. The goal achievement scenario was assumed that the goal of the number of wooden houses to be built in FY2030 (10,000 houses/yr)^55^ will be achieved by the target company, and it will continue with the same increasing trend until FY2050. For both scenarios, the parameters including half-life until FY2050 for each lifetime function were set to remain unchanged from those until FY2020.

## Supplementary Information

Supplementary Information.

## Data Availability

The datasets generated and analyzed during the current study are available from the corresponding author on reasonable request.
