# Supplementary material for: Estimation of carbon stocks in wood products for private building companies
Source: Sci Rep. 2022 Oct 27;12:18112. doi: 10.1038/s41598-022-23112-0 (PMC9613633; doi:10.1038/s41598-022-23112-0)
Supplement: Supplementary file 1 — Supplementary Information. [file 41598_2022_23112_MOESM1_ESM.docx]

**Supplementary Information**

**Table S1.** Number of wooden houses and average floor area per house built by the target company.

| FY | (1) | (2) | (3) | FY | (1) | (2) | (3) |
| --- | --- | --- | --- | --- | --- | --- | --- |
| 1969 | 1 | 1 | 81 | 1995 | 10784 | 10581 | 152 |
| 1970 | 18 | 18 | 81 | 1996 | 11838 | 11683 | 154 |
| 1971 | 43 | 43 | 84 | 1997 | 10672 | 10568 | 156 |
| 1972 | 47 | 46 | 80 | 1998 | 9523 | 9444 | 155 |
| 1973 | 55 | 53 | 75 | 1999 | 11003 | 10926 | 154 |
| 1974 | 25 | 24 | 79 | 2000 | 11306 | 11246 | 154 |
| 1975 | 41 | 40 | 83 | 2001 | 10404 | 10349 | 151 |
| 1976 | 142 | 127 | 88 | 2002 | 10150 | 10122 | 149 |
| 1977 | 269 | 241 | 98 | 2003 | 10088 | 10052 | 147 |
| 1978 | 511 | 470 | 108 | 2004 | 9984 | 9962 | 146 |
| 1979 | 866 | 797 | 108 | 2005 | 9695 | 9671 | 144 |
| 1980 | 1138 | 1027 | 104 | 2006 | 9580 | 9554 | 145 |
| 1981 | 1234 | 1141 | 104 | 2007 | 9401 | 9378 | 145 |
| 1982 | 1625 | 1514 | 108 | 2008 | 9458 | 9446 | 141 |
| 1983 | 1679 | 1561 | 146 | 2009 | 8885 | 8876 | 137 |
| 1984 | 2574 | 2314 | 123 | 2010 | 9090 | 9080 | 136 |
| 1985 | 3059 | 2795 | 128 | 2011 | 9420 | 9408 | 135 |
| 1986 | 3783 | 3474 | 125 | 2012 | 9475 | 9467 | 135 |
| 1987 | 4339 | 4122 | 125 | 2013 | 9839 | 9838 | 135 |
| 1988 | 5448 | 5225 | 175 | 2014 | 9401 | 9396 | 135 |
| 1989 | 6385 | 6170 | 146 | 2015 | 8795 | 8794 | 134 |
| 1990 | 7103 | 6915 | 149 | 2016 | 8924 | 8922 | 134 |
| 1991 | 7748 | 7561 | 151 | 2017 | 8364 | 8363 | 131 |
| 1992 | 8607 | 8402 | 150 | 2018 | 8303 | 8301 | 129 |
| 1993 | 9590 | 9406 | 150 | 2019 | 8132 | 8132 | 128 |
| 1994 | 10342 | 10178 | 152 | 2020 | 8019 | 8019 | 126 |

(1) Number of wooden houses built in each FY between FY1969 and FY2020 (unit: house/yr).

(2) Number of existing wooden houses built in each FY between FY1969 and FY2020 at the start of FY2021 (unit: house).

(3) Average floor area per house built in each FY between FY1969 and FY2020 (unit: m^2^/house).

**Table S2.** Amount of wood products for each tree species used in wooden houses built by the target company (unit: m^3^/yr).

| Tree species/wood product category | FY2018 | FY2019 |
| --- | --- | --- |
| Japanese cedar | 22031 | 20680 |
| Hinoki cypress | 9551 | 9060 |
| Japanese red pine | 1971 | 2054 |
| Japanese larch | 2701 | 1805 |
| Sakhalin fir | 1122 | 1335 |
| Yezo spruce | 50 | 1 |
| Douglas fir | 309 | 353 |
| Western hemlock | 78 | 60 |
| Norway spruce | 130 | 158 |
| Sequoia sempervirens | 57 | 103 |
| Mixture of Norway spruce and Sequoia sempervirens | 81784 | 78977 |
| Mixture of Douglas fir and Dahurian larch | 1360 | 1470 |
| Mixture of lodgepole pine, white spruce, and noble fir | 4650 | 3575 |
| Plywood, wood board, laminated veneer lumber | 103875 | 109711 |
